# Data acquisition system for chemical kinetic studies

**DOI:** 10.1155/S1463924689000258

**Published:** 1989

**Authors:** Yu-zhen Zhu, Xin Zhou, Xiang-sheng Zang

**Affiliations:** 1 Department of Chemistry East China University of Chemical Technology Shanghai China; 2 Materials Science Department Shanghai Jiao Tong University Shanghai 200 030 China

## Abstract

A microcomputer-interfaced data acquisition system for chemical kinetics (interfacing with laboratory analogue instruments) has been developed. Analogue signals from instruments used in kinetics experiments are amplifed by a wide-range adjustable high-gain operational amplifier and smoothed by an op-based filter, and then digitized at rates of up to 10^4^ samples per channel by an ADC 0816 digitizer. The ADC data transfer and manipulation routine was written in Assembler code and in high-level language; the graphics package and data treatment package is in Basic. For the various sampling speeds, all of the program can be written using Basic-Assembler or completely in Assembler if a high sampling rate is needed. Several numerical treatment methods for chemical kinetics have been utilized to smooth the data from experiments.

The computer-interfaced system for second-order chemical kinetic studies was applied to the determination of the rate constant of the saponification of ethyl acetate at 35°C. For this specific problem, an averaging treatment was used which can be called an interval method. The use of this method avoids the diffcully of measuring the starting time of the reaction. Two groups of experimental data and results were used to evaluate the systems performance. All of the results obtained are in agreement with the reference value.


Journal of Automatic Chemistry Vol. 11, No. 3 (May–June 1989), pp. 113–118

### Data acquisition system for chemical kinetic studies

**Yu-zhen Zhu, Xin Zhou* and Xiang-sheng Zang**
*Department of Chemistry, East China University of Chemical Technology, Shanghai, People's Republic of China*

*A microcomputer-interfaced data acquisition system for chemical kinetics (interfacing with laboratory analogue instruments) has been developed. Analogue signals from instruments used in kinetics experiments are amplified by a wide-range adjustable high-gain operational amplifier and smoothed by an op-based filter, and then digitized at rates of up to $10^4$ samples per channel by an ADC 0816 digitizer. The ADC data transfer and manipulation routine was written in Assembler code and in high-level language; the graphics package and data treatment package is in BASIC. For the various sampling speeds, all of the program can be written using BASIC–Assembler or completely in Assembler if a high sampling rate is needed. Several numerical treatment methods for chemical kinetics have been utilized to smooth the data from experiments.*

*The computer-interfaced system for second-order chemical kinetic studies was applied to the determination of the rate constant of the saponification of ethyl acetate at 35°C. For this specific problem, an averaging treatment was used which can be called an interval method. The use of this method avoids the difficulty of measuring the starting time of the reaction. Two groups of experimental data and results were used to evaluate the systems performance. All of the results obtained are in agreement with the reference value.*

### Introduction

In recent years, work has continued in applying microcomputers to chemical problems. Computers were initially used in chemistry for numerical manipulations in chemical problems, but microcomputers are now used for data acquisition and control and for data processing [1–3]. As the chemical problems may be complicated and the interfaces may vary between different types of microcomputers and instruments, work on microcomputer-based systems for particular chemical problems and chemical transducer interfacing is very important. In addition, in most laboratories it is necessary to interface microcomputers with traditional analogue instruments to create digital systems.

For the study of chemical kinetics, suitable instruments are needed to monitor signals from the physical variables in the reaction systems both rapidly and accurately. Any quantity which can represent the physical parameters in reaction and then be converted to a voltage can be measured by a microcomputer which is suitably interfaced to a chemical sensor or chemical transducer (for example, a pH meter, electrical conductivity meter, spectrophotometer or electrode). The advantages of microcomputer-based systems are that manual effort is reduced, the quality and amount of data obtained are improved and it is possible to process large amounts of data. It was these advantages that led the authors to design a microcomputer-interfaced system to meet the needs of data acquisition and numerical analysis in chemical kinetic studies.

This paper describes an 8-bit microcomputer, based on a Z-80 processor, which is interfaced to a laboratory-made conductivity meter and to other instruments commonly used for chemical kinetic studies. The system has been used to determine the rate constant of the saponification of ethyl acetate, with satisfactory results.

### System overview

### *Hardware*

Figure 1 shows a schematic diagram of the set-up; it consists of a MIC-80 microcomputer with 48K of RAM (which is compatible with a Radio Shack TRS-80), a C-60 tape-recorder, a line-printer and an *X–Y* plotter, interfaced to a conductivity meter (Model DDS-11A; Shanghai No. 2 Analytic Instrument Manufactory, Shanghai, China); a pH meter and other instruments can also be connected. The DDS-11A electrical conductivity meter is an analogue instrument that is still in routine use in China. It generates an analogue voltage, as a function of electrical conductance, which is indicated by a panel, analogue meter. The analogue voltage signal is obtained from an external socket in the range 0–10 mV full-scale.

The signal from the conductivity meter should be pre-processed by an analogue device to allow maximum resolution in the A/D converter and to remove high-frequency noise. Figure 2 illustrates a three-stage analogue circuit that performs these functions. The first stage acts as a simple high-gain inverting amplifier, which has variable resistive elements in the feedback loops, so that the amplification can be changed over a wide range to adapt different A/D input ranges to the instruments. The second stage provides an adjustable zero offset. According to accepted sampling theory, a signal must be sampled at a rate greater than twice the maximum frequency present, in order to avoid aliasing. It is also necessary to filter the signal obtained. The third stage of the circuit limits high frequencies via a low-pass filter. On leaving the filter the voltage, which is in 0–5·12 V full-scale range, is employed for interfacing and data logging by the MIC-80 through the A/D converter.

* *Author to whom correspondence should be addressed. Present address: Materials Science Department; Shanghai Jiao Tong University, Shanghai 200 030, People's Republic of China.*

0142–0453/89 $3.00 © 1989 Taylor & Francis Ltd.

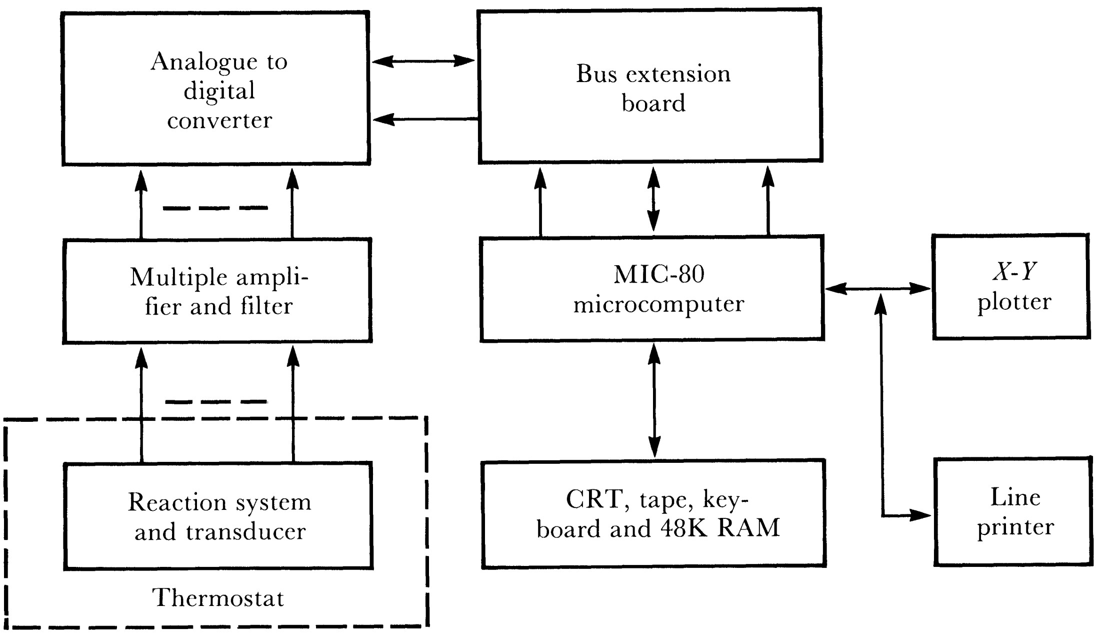

*Figure 1. Flow diagram of microcomputer-interfaced chemical kientics system.*

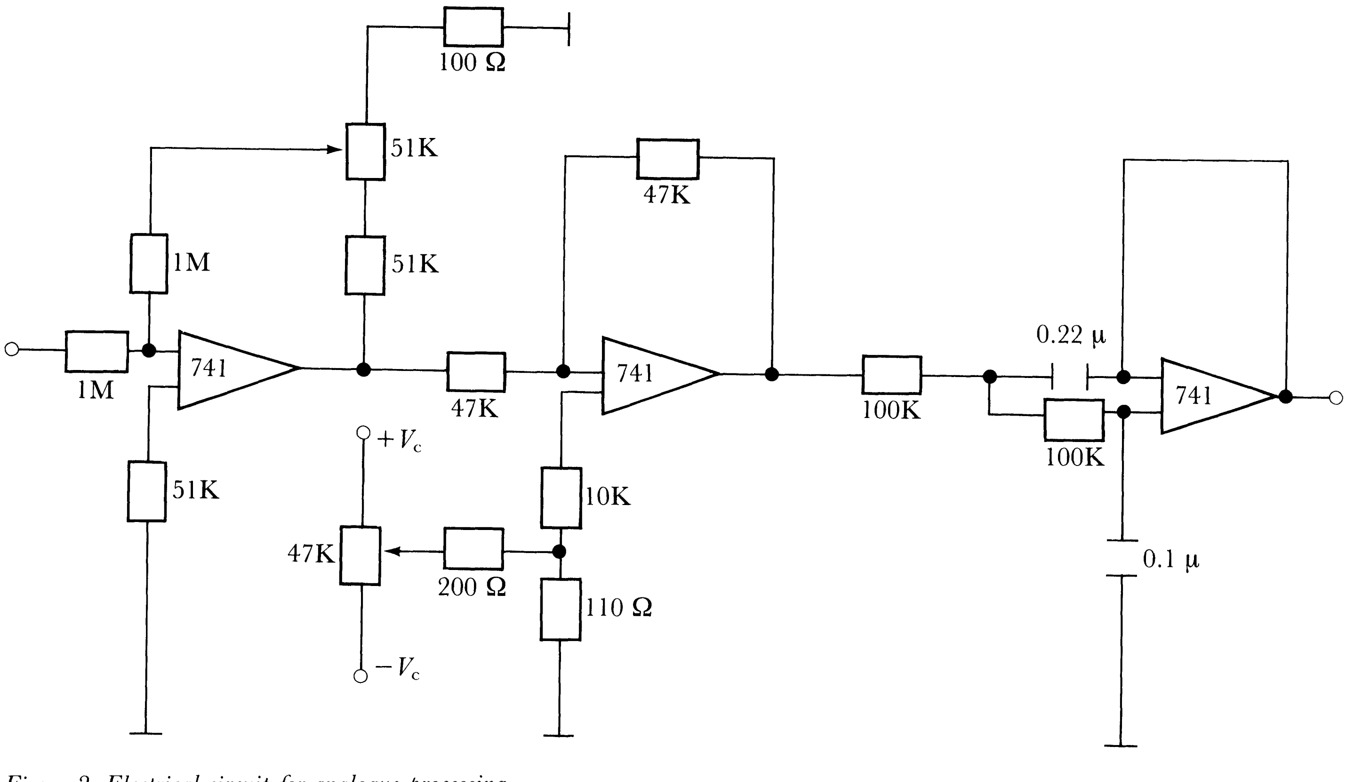

*Figure 2. Electrical circuit for analogue processing.*

The A/D converter is a Model ADC 0816 device (National Semiconductor, USA). It converts voltages between 0 and 5·12 V into an 8-bit binary number in 100 μs. The device has a 16-channel input multiplexer (which is excessive if the A/D converter is dedicated to the conductivity meter). Figure 3 shows a schematic diagram of the A/D converter; it clears on a high and starts a conversion on the falling edge of a TTL signal input to the START pin. Channel selection is also performed by the pulse to the ALE pin (see figure 3).

When the computer enables *OUT* and sets the address of the chip selection of the A/D converter and the channel number on the data bus simultaneously, START and ALE signals are generated and channel selection information is strobed into the A/D converter. After a 100-μs delay for its conversion, the EOC signal goes high, to indicate that data transfer is permitted. This transfer from the internal data latch of the A/D converter to the microcomputer is accomplished by enabling *IN*. Assuming that the address of the chip selection is 253 (FD in

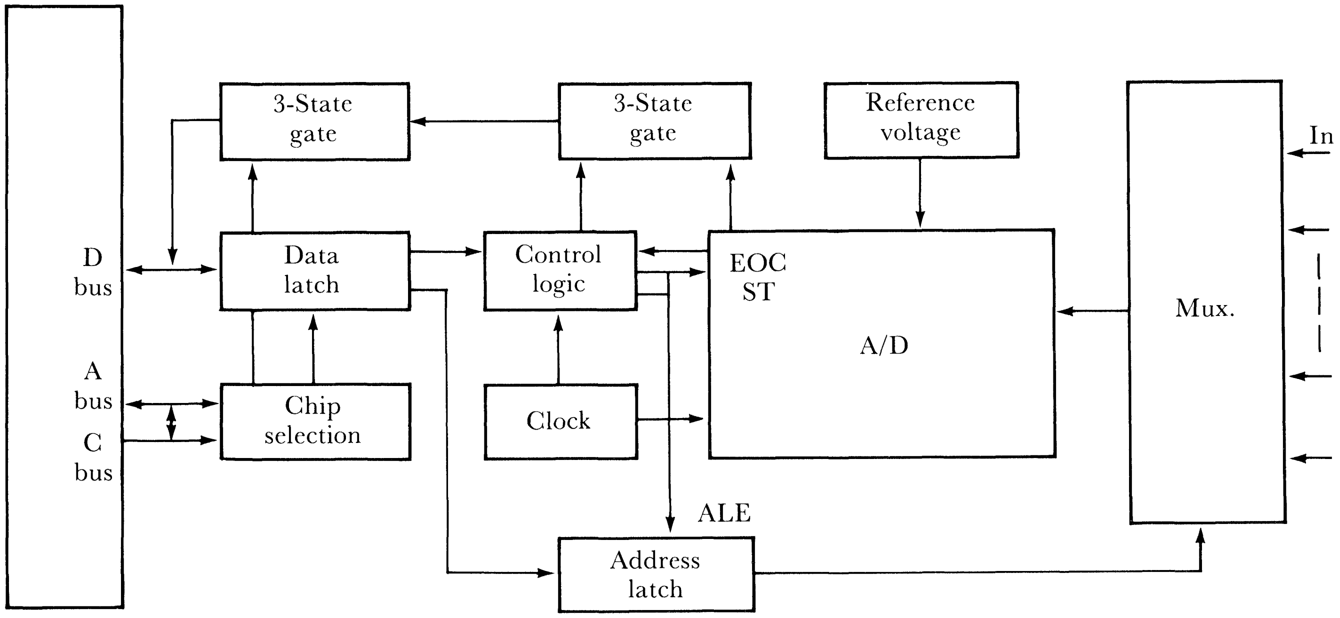

*Figure 3. Flow diagram of A/D converter*

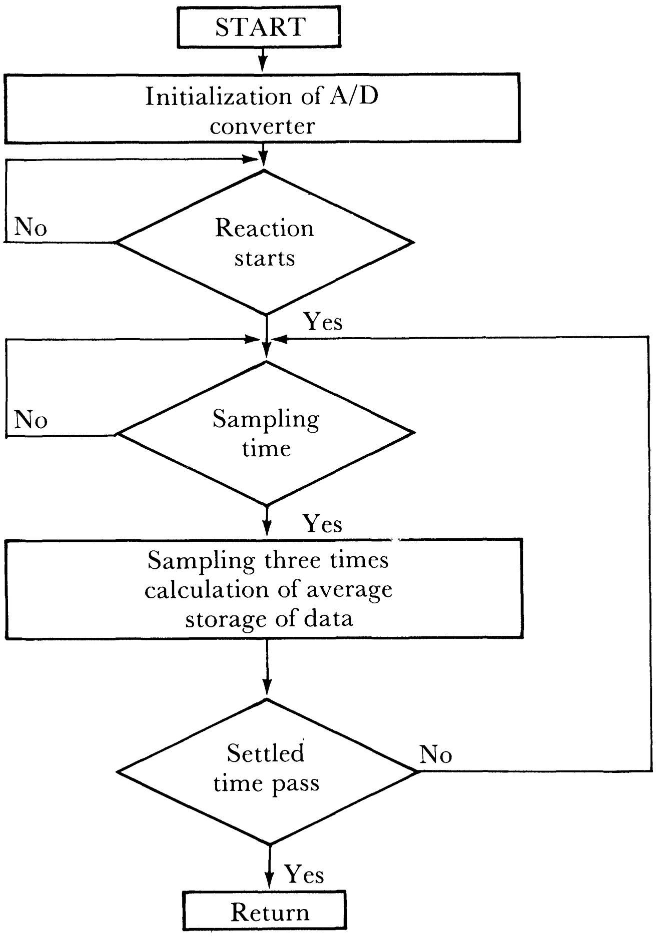

*Figure 4. Flowchart of sampling data.*

binary), channel 1 is selected; to start the A/D converter the following statements are used: OUT 253, 1, in extended BASIC or OUT (0FD), A in Assembler. To load data, use X=INP(253) or IN A, (0FD).

The maximum sampling frequency of the ADC 0816 is up to 0·625 kHz when all 16 channels are utilized. A 10-kHz sampling rate is obtainable when only one channel is in use.

*Software*

All real-time operations are directed by the MIC-80 via software commands. The MIC-80 microcomputer can be programmed both in BASIC and in Assembler. However, BASIC has a USR functional statement which provides a link to assembly language sub-routines. As many chemists have no experience in assembly language programming, the most convenient tool for programming is high-level languages such as BASIC or PASCAL. However, in some instances a high speed in sampling data is required, and using the BASIC sub-routine to control the A/D converter cannot meet this requirement. To solve this problem, the BASIC-Assembler method can be used. Whenever required, the Assembler sub-routine is called with a USR functional statement in BASIC. An increase in the data transfer rate to the MIC-80 is possible by using this approach or the Assembler only method.

The software can be divided into several blocks according to the functions:

(1) A main routine in BASIC is responsible for the management of all Assembler or BASIC sub-routines, programming for specific chemical problems, output of results, display of data and interaction with chemists.

(2) An Assembler routine which controls the A/D converter and provides high-speed transfer of the binary data into the MIC-80. This sub-routine is also written in BASIC for slow reactions.

(3) A routine in BASIC and in Assembler that creates a delay time by controlling the software. This functions as a software timer.

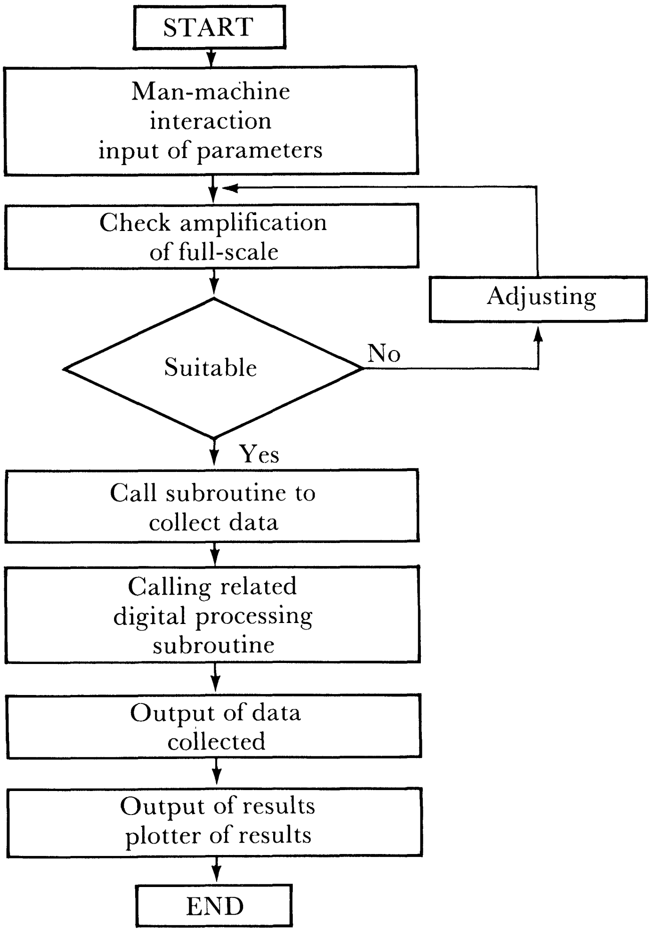

*Figure 5. Flowchart of main program.*

*Table 1. Data of determination of rate constant of saponification of ethyl acetate, where* $t'(j) = \Delta t_j$, $g(i) = G_{t_i}$. Parameters: CH = 0·04385; OH = 0·01014; $G_0$ = 2·99; Gc = 1·10331; and $T$ = 35 °C ± 0·5 °C.

| | | | |
|---|---|---|---|
| *Data set (a)*: | | | |
| $t'(0) = 0$ | $g(0) = 2{\cdot}46$ | $Y(0) = 1{\cdot}73$ | $K(0) = 0$ |
| $t'(1) = 0{\cdot}5$ | $g(1) = 2{\cdot}19$ | $Y(1) = 0{\cdot}18$ | $K(1) = 10{\cdot}68$ |
| $t'(2) = 1$ | $g(2) = 1{\cdot}98$ | $Y(2) = 0{\cdot}37$ | $K(2) = 10{\cdot}98$ |
| $t'(3) = 1{\cdot}5$ | $g(3) = 1{\cdot}79$ | $Y(3) = 0{\cdot}59$ | $K(3) = 11{\cdot}67$ |
| $t'(4) = 2$ | $g(4) = 1{\cdot}68$ | $Y(4) = 0{\cdot}74$ | $K(4) = 10{\cdot}98$ |
| $t'(5) = 2{\cdot}5$ | $g(5) = 1{\cdot}58$ | $Y(5) = 0{\cdot}92$ | $K(5) = 10{\cdot}92$ |
| $t'(6) = 3$ | $g(6) = 1{\cdot}46$ | $Y(6) = 1{\cdot}19$ | $K(6) = 11{\cdot}77$ |
| $t'(7) = 3{\cdot}5$ | $g(7) = 1{\cdot}41$ | $Y(7) = 1{\cdot}34$ | $K(7) = 11{\cdot}36$ |
| $t'(8) = 4$ | $g(8) = 1{\cdot}34$ | $Y(8) = 1{\cdot}58$ | $K(8) = 11{\cdot}72$ |
| $t'(9) = 4{\cdot}5$ | $g(9) = 1{\cdot}29$ | $Y(9) = 1{\cdot}81$ | $K(9) = 11{\cdot}93$ |
| $t'(10) = 5$ | $g(10) = 1{\cdot}27$ | $Y(10) = 1{\cdot}92$ | $K(10) = 11{\cdot}39$ |
| $t'(11) = 5{\cdot}5$ | $g(11) = 1{\cdot}22$ | $Y(11) = 2{\cdot}27$ | $K(11) = 12{\cdot}24$ |
| $t'(12) = 6$ | $g(12) = 1{\cdot}21$ | $Y(12) = 2{\cdot}36$ | $K(12) = 11{\cdot}67$ |
| $K = 11{\cdot}44$ | | | |
| *Data set (b)*: | | | |
| $t'(0) = 0$ | $g(0) = 2{\cdot}31$ | $Y(0) = 1{\cdot}82$ | $K(0) = 0$ |
| $t'(1) = 0{\cdot}5$ | $g(1) = 2{\cdot}08$ | $Y(1) = 0{\cdot}18$ | $K(1) = 10{\cdot}68$ |
| $t'(2) = 1$ | $g(2) = 1{\cdot}87$ | $Y(2) = 0{\cdot}40$ | $K(2) = 11{\cdot}87$ |
| $t'(3) = 1{\cdot}5$ | $g(3) = 1{\cdot}73$ | $Y(3) = 0{\cdot}58$ | $K(3) = 11{\cdot}47$ |
| $t'(4) = 2$ | $g(4) = 1{\cdot}62$ | $Y(4) = 0{\cdot}76$ | $K(4) = 11{\cdot}27$ |
| $t'(5) = 2{\cdot}5$ | $g(5) = 1{\cdot}52$ | $Y(5) = 0{\cdot}96$ | $K(5) = 11{\cdot}39$ |
| $t'(6) = 3$ | $g(6) = 1{\cdot}44$ | $Y(6) = 1{\cdot}16$ | $K(6) = 11{\cdot}47$ |
| $t'(7) = 3{\cdot}5$ | $g(7) = 1{\cdot}38$ | $Y(7) = 1{\cdot}34$ | $K(7) = 11{\cdot}36$ |
| $t'(8) = 4$ | $g(8) = 1{\cdot}31$ | $Y(8) = 1{\cdot}63$ | $K(8) = 12{\cdot}09$ |
| $t'(9) = 4{\cdot}5$ | $g(9) = 1{\cdot}29$ | $Y(9) = 1{\cdot}72$ | $K(9) = 11{\cdot}34$ |
| $t'(10) = 5$ | $g(10) = 1{\cdot}25$ | $Y(10) = 1{\cdot}96$ | $K(10) = 11{\cdot}63$ |
| $t'(11) = 5{\cdot}5$ | $g(11) = 1{\cdot}22$ | $Y(11) = 2{\cdot}18$ | $K(11) = 11{\cdot}76$ |
| $t'(12) = 6$ | $g(12) = 1{\cdot}20$ | $Y(12) = 2{\cdot}37$ | $K(12) = 11{\cdot}72$ |
| $K = 11{\cdot}50$ | | | |

(4) A Basic routine library that can process the data resulting from various numerical treatments, such as smoothing, digital data filtering and least-squares treatment.

(5) An output routine in Basic that drives the $X$–$Y$ plotter.

A flow chart of the software for controlling the A/D converter and data is shown in figure 4.

In kinetic experiments it is difficult to achieve satisfactory signal-to-noise ratios because analogue instruments are highly sensitive, and smoothing and differentiating experimental data are therefore very important. Methods that the authors have used successfully include least-squares polynomial filters and the signal moving-average method [4]. In addition to other advantages, the least-squares method does not require a regular interval between the data collected, so it is useful for chemical kinetic studies to smooth data. Another common method for smoothing data is Fourier transformation [5] and the microcomputer-interfaced system makes this method easier for chemists to use.

### Experimental

The experiment performed to evaluate the performance characteristics of the system was the determination of the rate constant of the saponification of ethyl acetate by electrical conductivity measurements [6].

All reagents were of analytical-reagent grade. The solutions for the kinetic experiment were kept in temperature-controlled store. The conductivity of the reaction system was measured with a DDS-11A meter connected to the MIC-80. This reaction is briefly described as follows:

$$CH_3COOC_2H_5 + OH^- \rightarrow CH_3COO^- + C_2H_5OH$$

$$t = 0: \quad CH \qquad OH \qquad 0$$

$$t = t: \quad CH - x \qquad OH - x \qquad x$$

where $x$ is the number of moles per litre at time $t$, CH is the initial molar concentration of $CH_3COOC_2H_5$ and OH is that of $OH^-$.

It has been found that the reaction follows second-order kinetics, so we can obtain by integration of its velocity equation:

$$\ln\left[\frac{(CH - x)OH}{(OH - x)CH}\right] = K(CH - OH)t \qquad (1)$$

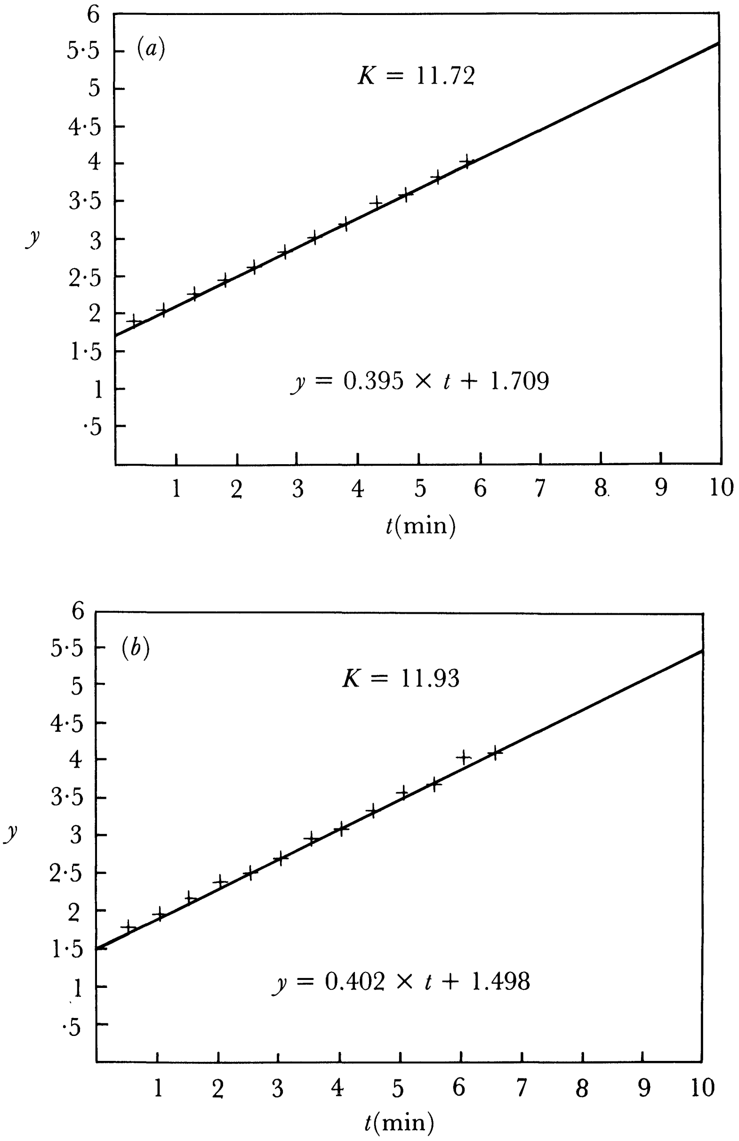

*Figure 6.* (a) *Least-squares plot for data set* (a) *of table 1*; (b) *least-squares plot for data set* (b).

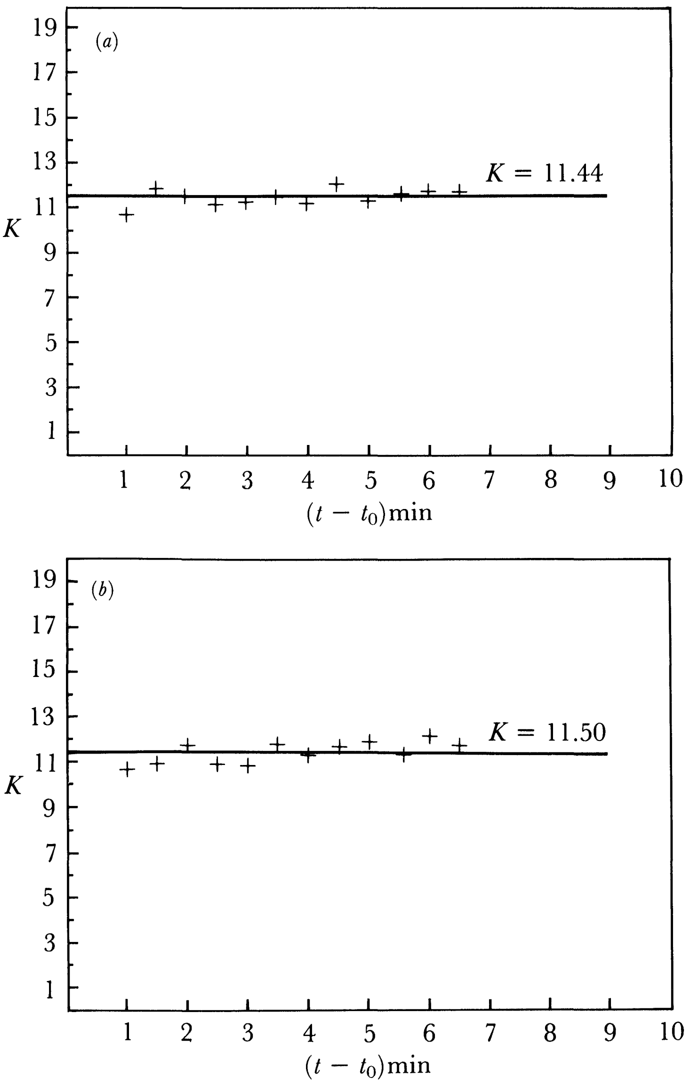

*Figure 7.* (a) *Plot of average method for data set* (a) *of table 1*; (b) *plots of average method for data set (b).*

From the relationship between conductivity and $OH^-$, $CH_3COO^-$ and $Na^+$, $x$ is given by

$$\frac{x}{C}=\frac{G_0-G_t}{G_0-G_c} \tag{2}$$

Where $G_t$ is the conductance of the solution at time $t$, $G_c$ that of the solution at time $t = \infty$ and $G_0$ that at time $t = 0$. By rearranging the above equations, we obtain

$$\ln\left[\frac{(G_0-G_t)\mathrm{CH}-(G_0-G_t)C}{(G_0-G_c)\mathrm{OH}-(G_0-G_t)C}\right] = K(\mathrm{CH}-\mathrm{OH})t+\ln\left(\frac{\mathrm{CH}}{\mathrm{OH}}\right) \tag{3}$$

To calculate the value of $K$ from the data collected, both the least-squares and average methods can be used. The average method is treated as follows: for $n + 1$ pairs of data $(t_i, G_{ti})$, $i = 0, 1, 2, \ldots, n$, let $\Delta t_j = t_i - t_0$. The value of $K$ can then be derived:

$$K_j=\frac{\ln\left[\frac{(G_0-G_c)\mathrm{CH}-(G_0-G_{t_j})C}{(G_0-G_c)\mathrm{OH}-(G_0-G_{t_j})C}\right]-\ln\left[\frac{(G_0-G_c)\mathrm{CH}-(G_0-G_{t_0})C}{(G_0-G_c)\mathrm{OH}-(G_0-G_{t_0})}\right]}{(\mathrm{CH}-\mathrm{OH})t_j} \tag{4}$$

$$\bar{K}=\frac{1}{n}\sum_{j=1}^{n}K_j$$

where $C = \min(\mathrm{OH}, \mathrm{CH})$.

The average method used here deals only with the time interval and corresponding electrical conductivity in order to avoid the difficulty of measuring the starting time of the reaction exactly. The least-squares method is treated by letting $Y$ equal the left-hand side of equation (3) through linear regression against variable $t$.

Using the average method and least-squares treatment to study the reaction, the experiment was performed several times. The results show that the rate constant obtained is in agreement with the results of previous studies [6]. Table 1 gives the two groups of experimental data. Figures 6 and 7 include parts of the results given by the $X$–$Y$ plotter using the average method and least-squares treatment.

### Conclusion

This microcomputer-interfaced system could prove useful in chemical kinetic studies as it can control several commonly used chemical instruments. All processing from data logging and storage to final data processing, display and printing can now be performed automatically in the laboratory. Data acquisition can be achieved at higher rates and is more accurate than with traditional methods.

Although the microcomputer used was a MIC-80, the technique is equally applicable to other microcomputers.

### Acknowledgements

The author thanks Mr Xiong Xiang for valuable discussions and also acknowledges the assistance given by Mr Zhang Lizhung.

### References

1. Venter, B. H., and Siebert, M. L., *Journal of Automatic Chemistry*, **7** (1985), 80.
2. Rossi, D. T., and Pardue, H., *Journal of Automatic Chemistry*, **7** (1985), 64.
3. Stanley, R., *Journal of Automatic Chemistry*, **6** (1984), 6.
4. Khan, A., *Analytical Chemistry*, **59** (1987), 654.
5. Griffiths, P. R., in *Transform Techniques in Chemistry* (Plenum Press, New York, 1978), p. 254.
6. Daniels, F., *et al.*, in *Eperimental Physical Chemistry* (McGraw-Hill, New York, 1975), p. 144.

